# Multi‐omics analysis reveals insights into the differentiation between radiological subtypes of early lung adenocarcinoma

**DOI:** 10.1002/ctm2.70800

**Published:** 2026-08-28

**Authors:** Huiwen Miao, Ruiying Kong, Xiaobo Xu, Mimi Wang, Xiao Teng, Dingli Zhu, Wenyi Zhao, Sheng Cai, Jingqi Zhou, Xiayi Lv, Zhan Zhou

**Affiliations:** ^1^ Department of Thoracic Surgery The First Affiliated Hospital Zhejiang University School of Medicine Hangzhou China; ^2^ State Key Laboratory of Advanced Drug Delivery and Release Systems & Innovation Institute for Artificial Intelligence in Medicine School of Pharmacy Zhejiang University Hangzhou China; ^3^ Zhejiang Provincial Key Laboratory of Anti‐Cancer Drug Research & MOE Engineering Research Centre of Innovative Anticancer Drugs Zhejiang University Hangzhou China; ^4^ Department of HPB Surgery The First Affiliated Hospital Zhejiang University School of Medicine Hangzhou China; ^5^ Science and Education Information Section Hangzhou Centre for Disease Control and Prevention Hangzhou China; ^6^ School of Public Health Shanghai Jiao Tong University School of Medicine Shanghai China

Dear Editor,

Lung adenocarcinoma (LUAD) presenting as ground‐glass opacity (GGO) generally has a more favourable prognosis, often exhibiting indolent growth, whereas purely solid lesions are frequently associated with poorer clinical outcomes.^1^ Despite the disparity in clinical trajectories, the underlying molecular drivers dictating this divergence remain poorly understood. This can lead to controversies regarding appropriate surgical methods and potential overtreatment.^2^ Prior studies have identified transcriptome differences between GGO‐associated and solid LUAD, but the proteomic and metabolomic landscapes remain insufficiently characterised.^3^, ^4^ A comprehensive molecular characterisation of these distinct radiological phenotypes is therefore needed to identify biomarkers associated with tumour aggressiveness and clinical behaviour. To address this gap, we established a prospective cohort of 50 treatment‐naïve patients with stage T_1_N_0_M_0_ LUAD, including 26 GGO‐associated and 24 purely solid tumours, with partially paired adjacent normal tissues. Integrative profiling of the transcriptome, 4D‐DIA proteome and untargeted metabolome enabled us to construct a cross‐omics molecular atlas delineating the biological differences between these early‐stage LUAD subtypes (Figure 1 and Table S1).

**FIGURE 1:**
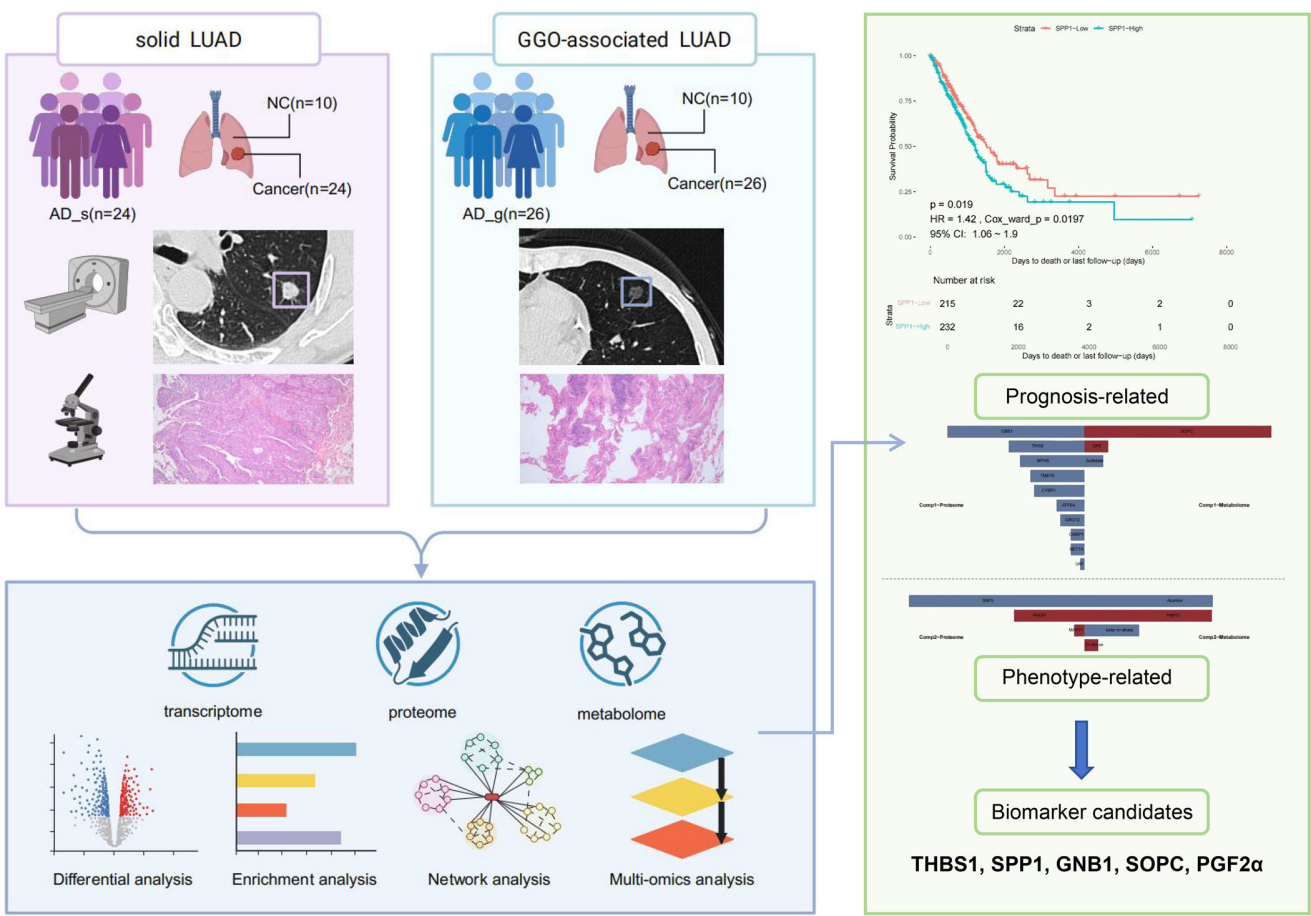
Research overview. Samples were collected based on the computed tomography (CT) images, and then multi‐omics analysis was conducted for comparison between the two groups. The CT images and pathological sections shown in the figure were typical examples from each of the two groups.

Proteomic profiling revealed a profound molecular divergence between the two radiological phenotypes, identifying an extracellular matrix (ECM)‐centric signature uniquely enriched in purely solid tumours. Specifically, 267 differentially expressed proteins (DEPs) distinguished the two subtypes, with a striking upregulation (*t*‐test *p* < 0.05 and |log_2_FoldChange| > 1) of pro‐invasive and tissue‐remodelling factors such as SPP1, MMP12 and THBS1 in solid lesions (Figure 2A–C and Figure S1). Beyond mere differential expression, protein‐protein interaction networks and WGCNA module analyses mapped a subtype‐related highly interconnected ECM‐associated functional hub. Within this interactome, THBS1 and SPP1 emerged as prominent components of the ECM‐associated proteomic signature enriched in solid LUAD (Figure 2D–J and Figures S2–S5). Biologically, these glycoproteins have been implicated in integrin signalling, focal adhesion, and ECM remodelling, processes closely associated with tumour invasion and progression.^5^, ^6^ Consistent with their robust roles in driving malignancy, our clinical validation using the TCGA‐LUAD external cohort revealed that high expression of THBS1 (*p* = 0.0108, hazard ration [HR] = 1.54, 95% confidence interval [95% CI]: 1.06–1.90) and SPP1 (*p* = 0.0190, HR = 1.42, 95% CI: 1.10–2.14) significantly correlates with shortened overall survival. These findings support the prognostic relevance of ECM‐associated markers identified in our proteomic analysis.

**FIGURE 2:**
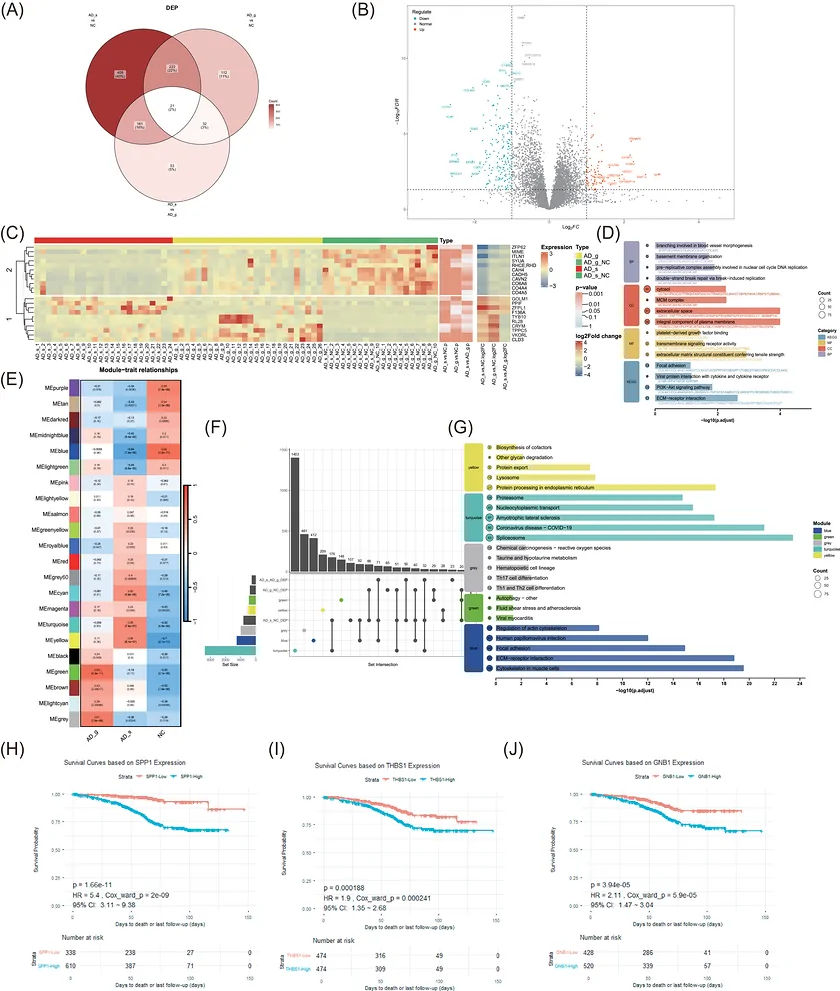
Proteomic comparative analysis of lung adenocarcinoma subtypes. (A) Venn diagrams showing the number and overlap of differentially expressed proteins (DEPs). (B) Volcano plots illustrating differential protein expression between AD_g and AD_s. (C) Heatmap of the expression of DEPs (significant in all comparisons) across all groups. (D) Top 4 terms significantly changed in the comparison between AD_s and AD_g through GO and KEGG enrichment analysis. (E) Association of lung adenocarcinoma subtype with modules. (F) Upset plot of subtype‐related modules with DEPs across all the comparisons. (G) Enriched KEGG pathways of proteins in subtype‐related modules. Each module is annotated by its most significantly enriched pathway (colour labels are retained for visual consistency): blue, extracellular matrix (ECM) module (enriched for ECM–receptor interaction, focal adhesion, and regulation of actin cytoskeleton); turquoise, spliceosome/proteasome module (spliceosome, coronavirus disease–COVID‐19, amyotrophic lateral sclerosis, proteasome); yellow, ER protein processing module (protein processing in endoplasmic reticulum, lysosome, protein export); green, fluid shear stress and atherosclerosis module (viral myocarditis, autophagy–other); grey, immune differentiation module (Th1 and Th2 cell differentiation, Th17 cell differentiation, hematopoietic cell lineage). (H) Survival analysis of SPP1 (H), THBS1 (I) and GNB1 (J) using the LC‐1000 cohort.

The clinical relevance of these candidates was further supported by external validation. In an independent GGO‐stratified transcriptomic cohort,^4^ SPP1 (*p*_adj_ = 2.86 × 10^−71^), THBS1 (*p*_adj_ = 0.00012) and GNB1 (*p*_adj _= 2.86 × 10^−11^) showed significant subtype‐specific expression while higher expression of SPP1 (*p* = 1.66 × 10^−11^, HR = 5.4, 95% CI: 3.11–9.38), THBS1 (*p* = 1.88 × 10^−4^, HR = 1.90, 95% CI: 1.35–2.68) and GNB1 (*p *= 3.94 × 10^−5^, HR = 2.11, 95% CI: 1.47–3.04) were consistently associated with poorer overall survival across external LUAD datasets (Figure 2H–J, and Figures S6 and S7).

Untargeted metabolomic profiling functionally complemented the proteomic landscape, highlighting arachidonic acid metabolism as a critical metabolic differentiator. Variable importance in projection (VIP) scores derived from OPLS‐DA pinpointed key discriminatory metabolites (Figure 3A,B, and Figures S8 and S9). The top‐ranking features were arachidonic acid, oleic acid, and Cis‐4,7,10,13,16,19‐docosahexaenoic acid, which are all unsaturated fatty acids (Tables S3 and S4). Arachidonic acid metabolism was significantly altered (*p* = 0.043) between AD_s (adenocarcinoma, purely solid subtype) and AD_g (adenocarcinoma, GGO‐associated subtype). Enriched differential metabolites within this pathway included arachidonic acid‐related phospholipids and eicosanoids, and prostaglandin F2α (PGF2α) (Figure 3C–E and Figure S10).

**FIGURE 3:**
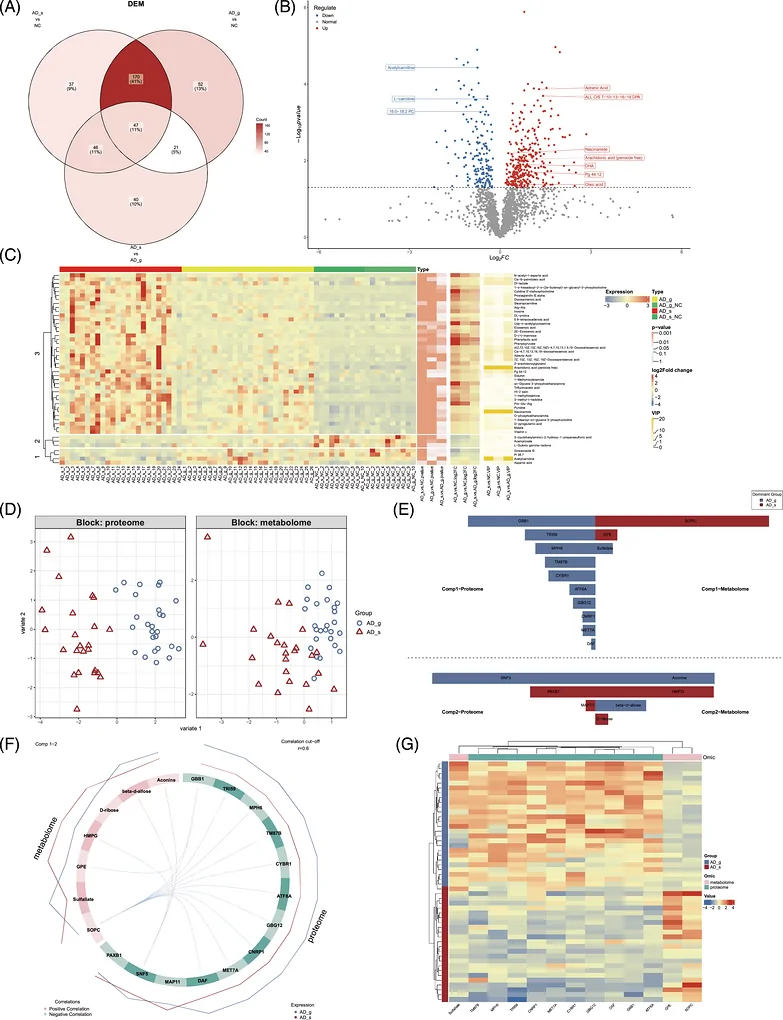
Comparative metabolomic analysis and integrated proteomics and metabolomics analysis of lung adenocarcinoma subtypes. (A) Venn diagrams showing the number and overlap of DEMs. (B) Volcano plots illustrating differential metabolites with the top 10 VIP scores between AD_g and AD_s. (C) Heatmaps of the expression of DEMs (between AD_s and AD_g) across all groups. (D) Sample projection onto the space spanned by the components of each block, coloured by lung adenocarcinoma subtype. (E) Loadings plot of contributing features using DIABLO, ranked by the absolute value of their coefficients. Colours indicate the category with the highest median expression for each feature. (F) Circos plot displaying positive (red lines) and negative (blue lines) correlations between selected multi‐omics features. (G) Clustering heatmap of multi‐omics features using Euclidean distance and complete linkage method, with rows representing samples and columns representing selected features on the first component.

Integrated omics analysis highlighted arachidonic acid metabolism as a key metabolic axis distinguishing the two LUAD subtypes. We constructed a Compound‐Reaction‐Enzyme‐Gene network, highlighting the arachidonic acid metabolism pathway as a key differentiator (Figures S11 and S12). DIABLO integration of proteomic and metabolomic data further demonstrated coordinated molecular differences between the two LUAD phenotypes, with GNB1 and SOPC among the discriminating features (Figure 3F–I).^7^ The resulting multi‐omics model achieved a clear separation (Figure 3F). The top discriminating features identified were the protein GNB1 (significantly lower in AD_s) and the metabolite SOPC (significantly higher in AD_s) (Figure 3G–I). Interestingly, GNB1 showed discordant transcriptomic and proteomic patterns, suggesting potential post‐transcriptional regulation. GNB1 encodes the G protein β1 subunit, which forms the Gβγ complex. Previous studies have demonstrated that Gβγ signalling can activate cytosolic phospholipase A2,^8^ promoting arachidonic acid release, suggesting a potential mechanistic link between GNB1‐mediated signalling and arachidonic acid metabolism, although this remains to be validated.

At the molecular level, our multi‐omics findings suggest that the arachidonic acid metabolic pathway is significantly altered in solid LUAD, accompanied by elevated levels of phospholipid metabolites and key enzymes (e.g. FADS2), as well as increased PGF2α levels (Figure 4A). This enhanced eicosanoid signalling is well‐documented to foster a localised inflammatory state and promote cancer progression.^9^ Previous reports have also indicated that the synthesis of PGF2α is elevated in lung cancer patients compared to normal tissues, and it can increase invasion and proliferation of cancer.^10^, ^11^ To assess the potential role of PGF2α, we evaluated its effect in a mouse tumour model. Administration of PGF2α at 3.0 mg/kg significantly accelerated tumour growth (Figure 4B), providing functional support for a tumour‐promoting role of the arachidonic acid‐eicosanoid axis.

**FIGURE 4:**
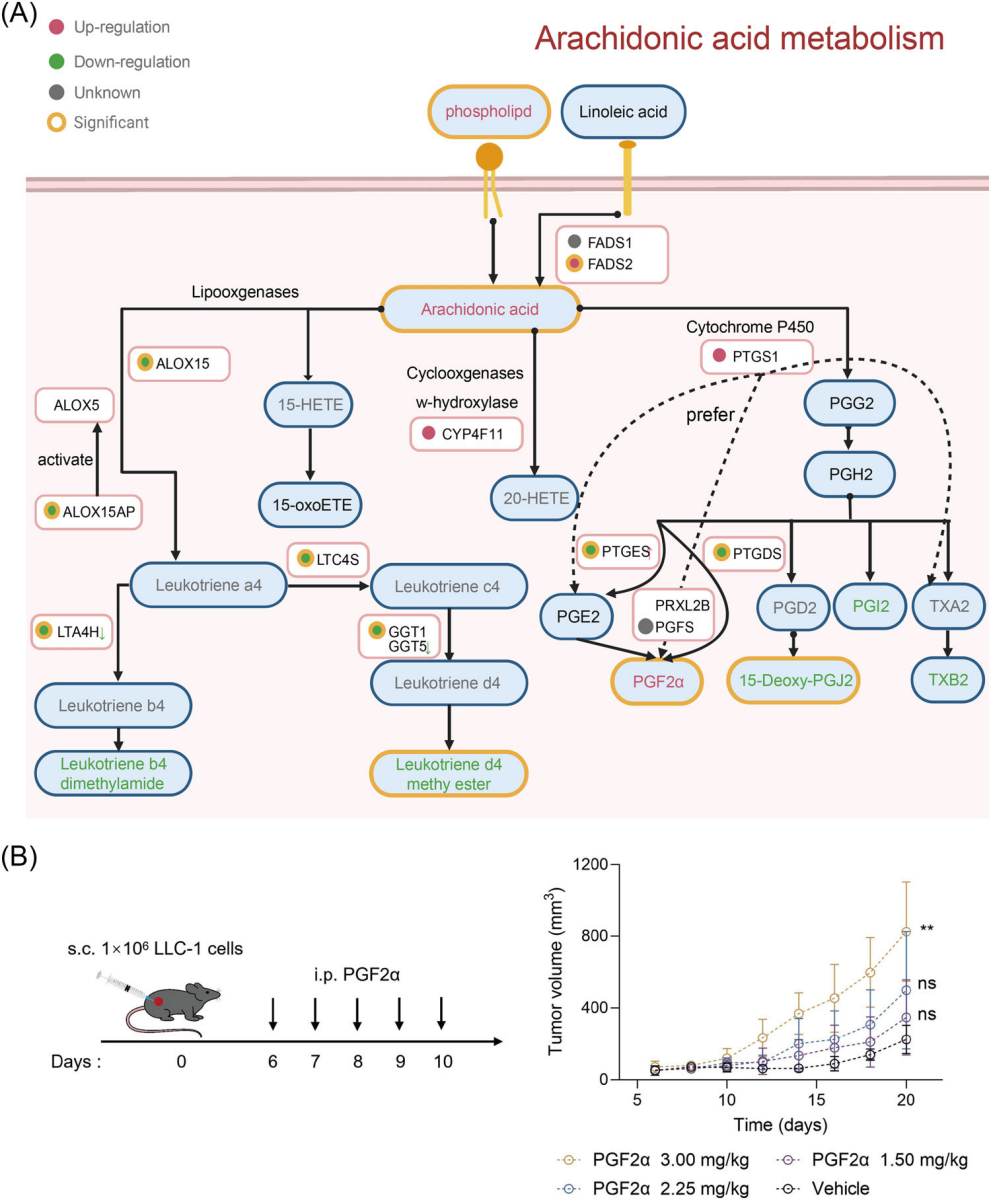
Protein and metabolite changes in the arachidonic acid metabolism pathway. (A) The fold changes (AD_s vs. AD_g) and significance of proteins and metabolites in the pathway are presented. Proteins or metabolites with no significant differences but with a fold change greater than 1.5 or less than 0.67 are also indicated in the figure. The line marked with “prefer” denotes that PTGS1 is preferentially (but not limited to) conjugated with PGE2, PGF synthase and TXA2. The red and green arrows beside the protein indicate that the gene is significantly upregulated and downregulated in the transcriptome. (B) Mouse metastasis tumour experiment with prostaglandin F2α (PGF2α). Female C57BL mice were subcutaneously injected with 10^6^ tumor cells. Six days after tumour formation, mice with comparable tumour sizes were selected. Prostaglandin tromethamine was administered by intraperitoneal injection every 2 days for a total of five injections.

Together, our findings reveal that purely solid LUAD is characterised by ECM remodelling and arachidonic acid‐eicosanoid metabolic reprogramming, distinguishing it molecularly from GGO‐associated disease. The convergence of proteomic, metabolomic, external transcriptomic, prognostic, and preliminary in vivo evidence further highlights SPP1/THBS1‐associated ECM remodelling and PGF2α signalling as candidate molecular features of aggressive early‐stage LUAD (Figures S13 and S14).

## AUTHOR CONTRIBUTIONS

Huiwen Miao, Xiayi Lv and Zhan Zhou conceived the study. Huiwen Miao, Xiaobo Xu, Mimi Wang and Xiao Teng collected tissue specimens and acquired omics data. Ruiying Kong, Dingli Zhu, Wenyi Zhao, Sheng Cai and Jingqi Zhou performed the analysis and interpreted the results. Ruiying Kong, Huiwen Miao, Jingqi Zhou and Zhan Zhou contributed to the writing of the manuscript draft. Jingqi Zhou, Xiayi Lv and Zhan Zhou supervised this study.

## FUNDING INFORMATION

This research was supported by the Zhejiang Provincial Natural Science Foundation of China (LTGY24H160010 and LQ24C060005), the “Pioneer and Leading Goose +X” S&T Program of Zhejiang (2025C02095) and the National Natural Science Foundation of China (32370712).

## CONFLICT OF INTEREST STATEMENT

The authors declare no conflict of interest.

## ETHICS STATEMENT

The study was approved by the Clinical Research Ethics Committee of the First Affiliated Hospital of Zhejiang University School of Medicine in accordance with the Declaration of Helsinki (2021 No. 663). Written informed consent was obtained from each patient.

## Supporting information



Supporting Information

## Data Availability

The raw transcriptome data reported in this paper have been deposited in the Genome Sequence Archive in the National Genomics Data Centre, China National Centre for Bioinformation/Beijing Institute of Genomics, Chinese Academy of Sciences (GSA‐Human: HRA012569), which is publicly accessible at https://ngdc.cncb.ac.cn/gsa‐human. Proteomic data have been deposited in NGDC OMIX (OMIX017893). Metabolomic data have been deposited in NGDC OMIX (OMIX017894).
