# Supplementary material for: Multi‐omics analysis reveals insights into the differentiation between radiological subtypes of early lung adenocarcinoma
Source: Clin Transl Med. 2026 Aug 28;16(9):e70800. doi: 10.1002/ctm2.70800 (PMC13522823; doi:10.1002/ctm2.70800)
Supplement: Supplementary file 1 [file CTM2-16-e70800-s001.docx]

# Supplementary Information

# 1. Materials and methods

**Tissue Specimen Collection and Study Design**

To study the two radiological phenotypes of LUAD, we collected tumor tissues and paired normal adjacent tissues from patients aged 18-70 years with early-stage LUAD (T_1_N_0_M_0_). Exclusion criteria included: 1) Postoperative pathological stage higher than Ⅰa. 2) Multiple primary lung cancer. 3) History of malignant tumors within 5 years. 4) History of chemotherapy. The study was approved by the Ethics Committee of the First Affiliated Hospital of Zhejiang University School of Medicine (2021 No. 663), and written informed consent was obtained from each patient. All patients underwent preoperative high-resolution CT scans to evaluate the lesions. According to whether the nodules contained ground-glass components or not, the samples were divided into the GGO-associated group (AD_g, n = 26) and pure solid group (AD_s, n = 24). Their partial paired normal adjacent tissues served as controls (NC, n=20).

**Proteomic Analysis**

4D-DIA Proteomic analysis (for 24 AD_s samples, 26 AD_g samples, 10 NC samples of AD_s and 10 NC samples of AD_g), wherein tissues were homogenized and sonicated ^1^. Lysates were centrifuged (14,000 g, 40 min), filtered (0.22 μm), and quantified via BCA Protein Assay Kit (Beyotime, P0012). Protein integrity was verified by SDS-PAGE (12.5%, Coomassie staining). After tryptic digestion, peptides were analyzed by nano-HPLC-MS/MS (Evosep One (Evosep, Denmark) coupled to timsTOF Pro (Bruker, Bremen, Germany), PASEF mode). DDA/DIA data were processed using Spectronaut (Biognosys AG, Switzerland, Uniprot_HomoSapiens_20387_20210928_9606_swissprot^2^, Carbamidomethyl (C) (fixed), Oxidation (M) and acetyl(Protein N-term) (variable), FDR ≤1%). Proteins showing |log_2_(FoldChange)| ≥ 1 and *P* < 0.05 (t-test) were considered expressed differentially. Blast2GO^3^ was used for GO annotation^4^ and KOALA ^5^ was used for KEGG pathway annotation.

In order to identify the key proteins among those differentially expressed proteins by integrating the known information on gene-gene interactions, we construct protein-protein interaction networks (PPI) between AD_g and AD_s with significant differences using the STRING (https://www.string-db.org/) database^6^, and the network construction results were imported into Cytoscape (v3.10.3)^7^ for visualization. To explore important proteins involved in phenotypic differences in lung adenocarcinoma, we use cytoHubba (v0.1)^8^ to calculate the 10 top-degree proteins as a sub-network, and MCODE (v2.0.2)^9^ was used to find important protein interaction modules. Community detection was performed using the igraph (v2.1.4)^10^ through edge centrality and random walk algorithms, and the sub-networks were annotated with KEGG using enrichR (v3.4)^11^.

WGCNA (v1.72-5) package in R was used to analyze modules^12^. After filtering down to the 5000 proteins with the highest median absolute deviation values, the data was input into weighted correlation network analysis (WGCNA) (corType = Pearson, networkType = unsigned, TOMType = unsigned, minModuleSize = 20, mergeCutHeight = 0.25, Power = 5).

**Metabolomics Analysis**

Metabolomic analysis (for 24 AD_s samples, 26 AD_g samples, 10 NC samples of AD_s and 10 NC samples of AD_g), wherein tissues were homogenized in H_2_O and methanol/acetonitrile (1:1, v/v), centrifuged (14,000 g, 4 °C, 15 min). Analysis was performed using a UHPLC (1290 Infinity LC, Agilent Technologies) coupled to a quadrupole time-of-flight (AB Sciex TripleTOF 6600) in Shanghai Applied Protein Technology Co., Ltd. For HILIC separation. HILIC column maintained at 25°C with a constant flow rate of 0.5 mL/min. The mobile phase consisted of (A) an aqueous solution containing 25 mM ammonium acetate and 25 mM ammonia, and (B) acetonitrile. A multi-step gradient elution protocol was implemented: initial conditions of 95% B (0-0.5 min), followed by linear reduction to 65% B (0.5-7 min), further decrease to 40% B (7-8 min), isocratic hold (8-9 min), rapid return to 95% B (9-9.1 min), and final re-equilibration (9.1-12 min). Samples were maintained at 4°C in the autosampler and analyzed in randomized order, with quality control samples interspersed throughout the analytical sequence to monitor system stability. Mass spectrometric detection was performed using an AB Triple TOF 6600 instrument operating in both positive and negative ESI modes with the following parameters: ion source gas1/2 (60 psi), curtain gas (30 psi), 600°C source temperature, and ±5500 V ion spray voltage. Full scan mass spectra (m/z 60-1000 Da) were acquired with 0.20 s/spectra accumulation time, while data-dependent MS/MS scans (m/z 25-1000 Da) used 0.05 s/spectra accumulation in high sensitivity mode. Collision-induced dissociation was performed at 35±15 eV with ±60 V declustering potential, employing dynamic exclusion of isotopic peaks (±4 Da) and monitoring up to 10 candidate ions per cycle. Raw data were converted to .mzXML using ProteoWizard, followed by peak alignment, retention time correction, and peak area extraction using XCMS^13,14^.

We then employed Orthogonal Partial Least Squares Discriminant Analysis (OPLS-DA) using ropls (v1.32.0)^15^ to establish a model for filtering out the noise that is unrelated to the group information to identify the important metabolites in the inter-group differences.

**Transcriptomic Analysis**

Transcriptomic analysis (for 10 AD_s samples, 10 AD_g samples and 10 NC samples), wherein total RNA was extracted from tissues. Sequencing libraries were generated using NEBNext® UltraTM RNA Library Prep Kit for Illumina® (NEB, USA) and index codes were added to attribute sequences to each sample^16^. 1 μg of total RNA from each sample underwent poly-A selection using magnetic beads, followed by chemical fragmentation in divalent cation buffer at elevated temperature. First-strand cDNA synthesis was performed with random hexamers and M-MuLV reverse transcriptase, while second-strand synthesis utilized DNA Polymerase I and RNase H. Following end blunting and adenylation of 3’ ends, NEBNext adapters were ligated. Size-selected fragments (250-300 bp) were selected through AMPure XP system (Beckman Coulter, Beverly, USA). Final library amplification employed Phusion High-Fidelity DNA polymerase with barcoded primers, with quality verification conducted on the Agilent Bioanalyzer 2100 system. Rigorous quality control was performed using an Agilent 2100 Bioanalyzer to assess RNA integrity. Pooled libraries were sequenced on an Illumina Novaseq platform using paired-end 150 bp reads. Clean reads were obtained by removing reads containing adapter, reads containing ploy-N and low quality reads from raw data. Filtered reads were aligned to the reference genome (GRCH38.p12) using HISAT2 (v2.0.5) with default parameters^17^. StringTie version 1.3.3b^18^ assembled mapped reads of each sample and featureCounts version 1.5.0-p3^19^ quantified gene expression levels. Sample reproducibility was evaluated using Pearson correlation coefficients and principal component analysis.

Raw counts were normalized via the median-of-ratios method, and significance thresholds were set at |log2(fold change)| ≥ 1 and adjusted p-value (padj) < 0.05^20^. Volcano plots and hierarchical clustering (Euclidean distance, complete linkage) were generated to visualize DEG patterns. Gene ontology (GO) terms and Kyoto Encyclopedia of Genes and Genomes (KEGG) pathways enrichment analyses based on hypergeometric distribution were conducted using clusterProfiler (v3.4.4)^21^.

**Multi-omics Integration Analysis**

DIABLO (mixOmics v6.26.0)^22^ was used to integrate proteomic and metabolomic analyses to identify key molecular factors from multi-omics analyses. After standardizing the raw data, through 10-fold cross validation repeated 10 times, the number of the components and the number of the parameters were optimized to get the final model. Contributions and associations of variables were mined for LUAD subtypes. Additionally, the differential proteins and differential metabolites were co-mapped to KEGG database pathways for metabolic pathway association analysis. Using metscape (v3.1.3)^23^, the transcriptome and metabolome data were used to establish a correlation network map for analysis to reveal the potential relationship between differentially expressed genes and differentially expressed metabolites.

**Multiplexed Immunohistochemistry**

Multiplexed immunohistochemistry (mIHC) was performed by staining 4-um-thick formalin-fixed, paraffin-embedded whole tissue sections with standard, primary antibodies sequentially and paired with TSA 6-color kit (M-D110061-50T,Yuanxibio). Then by staining with SN470. For example, Start the second to five round of staining，Slides were washed in TBST buffer and then transferred to preheated EDTA solution (100°C) before being heat-treated using a microwave set at 20% of maximum power for 15 minutes. Slides were cooled in the same solution to room temperature. incubated with anti-CD23 (#MA5-14572, Suppliers:Thermofisher, Lot:WI13374603) for 60 minutes and then treated with peroxidase-conjugated (HRP) secondary antibody (#DS9800, Leica) for 10 minutes. Then labelling was developed for a strictly observed 10 minutes, using TSA 620 per manufacturer’s direction. Between all steps, the slides were washed with Tris buffer. The same process was repeated for the following antibodies/fluorescent dyes, in order: anti-CD21 (#ab227662,Suppliers: abcam,Lot:GR3268546-6)/ TSA 570, anti-CD20 (#CM-0221, Suppliers:jiehaobio, Lot:CM02210322)/ TSA 670, anti-CD3 (#BX50022, Suppliers: biolynx, Lot:CPL703208P)/ TSA 520, anti-PanCK (#GM351507, Suppliers:Genetech, Lot:2022051501)/ TSA 440. Each slide was then treated with 2 drops of SN470 (A11010-100T; Yuanxibio), washed in distilled water, and manually coverslipped. Slides were air dried, and take pictures with Pannoramic MIDI tissue imaging system (3DHISTECH). Images was analyzed using Indica Halo software.

**Mouse metastasis tumor experiment with PGF2α**

C57BL mice, female, were subcutaneously injected with 1×10^6^ tumor cells. Six days after tumor formation, mice with comparable tumor sizes were selected. Prostaglandin tromethamine was administered by intraperitoneal injection every two days for a total of five injections.

# 2. Supplementary figures

## Figure S1 PCA of the identified proteins showing the difference between AD_s and AD_g.

## Figure S2 Differential protein community detection module with the top three KEGG annotations.

## Figure S3 22 modules in weighted correlation network analysis.

(A) PCA plot of the samples. (B) Selection of soft threshold in WGCNA. (C) Module hierarchical clustering tree of WGCNA.

## Figure S4 Kruskal-Wallis test and t-test for the expression of modules of different groups.

(A) Kruskal-Wallis test for the expression of modules of all the groups. (B) t-test for the expression of modules of different LUAD subtype.

## Figure S5 Hierarchical clustering heatmap of the blue module.

## Figure S6 Survival analysis conducted on patients with stage T_1_ in the TCGA-LUAD cohort. (A) SPP1 (HR = 1.94, *P* = 0.159) ; (B) THBS1 (HR = 1.56, *P* = 0.336); (C) GNB1 (HR = 0.57, *P* = 0.361).

## Figure S7 Survival analysis conducted on patients with stage T_1_N_0_M_0_ in the GSE30219 data. (A) SPP1 (HR = 2.00, *P* = 0.00314); (B) THBS1 (HR = 2.93, *P* = 0.00441); (C) GNB1 (HR = 1.74, *P* = 0.0479).

## Figure S8 OPLS-DA of the identified metabolites showing the difference between AD_s and AD_g.

## (A) OPLS-DA of the identified metabolites showing the difference between AD_s and AD_g in the positive ion mode. (B) OPLS-DA of the identified metabolites showed the difference between AD_s and AD_g in the negative ion mode.

## Figure S9 Permutation test plot of OPLS-DA analysis.

(A) Permutation test plot of AD_s vs NC model in the positive ion mode. (B) Permutation test plot of AD_g vs NC model in the positive ion mode. (C) Permutation test plot of AD_s vs AD_g model in the positive ion mode. (D) Permutation test plot of AD_s vs NC model in the negative ion mode. (E) Permutation test plot of AD_g vs NC model in the negative ion mode. (F) Permutation test plot of AD_s vs AD_g model in the negative ion mode.

## Figure S10 KEGG enrichment bubble plot of comparison between AD_s and AD_g.

## Figure S11 Compound-Reaction-Enzyme-Gene network of arachidonic acid metabolism pathway.

## Figure S12 Protein-protein interaction network visualized using Cytoscape.

## Highlighting the highest scoring modules identified by MCODE, marked in purple.

## Figure S13 Representative Masson-stained images and quantitative assessment of collagen deposition in an independent small cohort.

(A) Representative 10× Masson staining images from pure solid pulmonary nodules and ground-glass opacity (GGO) nodules, with the collagen-positive area percentage indicated for each sample. (B) Quantification of Masson collagen deposition. Bars represent mean ± SD, and dots indicate individual samples.

## Figure S14 Representative multiplex immunohistochemistry (mIHC) images of pulmonary nodules.

(A) Pure solid pulmonary nodule; (B) ground-glass opacity (GGO) nodule. Color code: CD21, green; CD20, yellow; CD23, red; CD3, purple; PanCK, cyan. Original images were proportionally scaled only; brightness, contrast, and colors were not adjusted.

# 3. Supplementary tables

## Table S1. Clinical characteristics

| Characteristic | Subtype | | *P* value |
| --- | --- | --- | --- |
|  | AD_s (n=24) | AD_g (n=26) |  |
| Age, year, mean (range) | 61 (41-75) | 56 (30-73) | 0.094 |
| Gender |  |  | 0.093 |
| Male | 12 (50.0%) | 7 (26.9%) |  |
| Female | 12 (50.0%) | 19 (73.1%) |  |
| Pathologic subtype |  |  | 0.0007 |
| APA | 8 (33.3%) | 11 (42.3%) |  |
| APA+IMA | 0 (0.0%) | 1 (3.8%) |  |
| PPA | 6 (25.0%) | 3 (11.5%) |  |
| SPA | 9 (37.5%) | 0 (0.0%) |  |
| IMA | 1 (4.2%) | 0 (0.0%) |  |
| LPA | 0 (0.0%) | 4 (15.4%) |  |
| MIA | 0 (0.0%) | 7 (26.9%) |  |
| Smoking |  |  | 0.024 |
| Yes | 13 (54.2%) | 6 (23.1%) |  |
| No | 11 (45.8%) | 20 (76.9%) |  |
| Tumor size, cm |  |  | 0.149 |
| ≤1 | 1 (4.2%) | 6 (23.1%) |  |
| 1-2 | 7 (29.2%) | 7 (26.9%) |  |
| 2-3 | 16 (66.7%) | 13 (50.0%) |  |
| Proportion of GGO components |  |  |  |
| <50% | / | 12 (46.2%) |  |
| >50% | / | 14 (53.8%) |  |

For continuous variables, *P* value is calculated using t-test; for categorical variables, *P* value is calculated using chi-square test.

## Table S2 Top 10 differentially expressed proteins in AD_s vs AD_g.

| ProteinNames | Genes | Fold change | P value | Adjusted P value |
| --- | --- | --- | --- | --- |
| GBB1 | GNB1 | 0.64 | 1.37×10^-13^ | 1.07×10^-9^ |
| TRI59 | TRIM59 | 0.61 | 1.37×10^-11^ | 5.32×10^-8^ |
| MPH6 | MPHOSPH6 | 0.69 | 8.84×10^-11^ | 2.29×10^-7^ |
| TM87B | TMEM87B | 0.63 | 1.63×10^-10^ | 3.04×10^-7^ |
| CYBR1 | CYBRD1 | 0.49 | 1.96×10^-10^ | 3.04×10^-7^ |
| ATF6A | ATF6 | 0.45 | 9.10×10^-10^ | 1.18×10^-6^ |
| GBG12 | GNG12 | 0.48 | 1.36×10^-9^ | 1.51×10^-6^ |
| CNRP1 | CNRIP1 | 0.53 | 3.47×10^-9^ | 3.37×10^-6^ |
| MET7A | METTL7A | 0.49 | 4.51×10^-9^ | 3.87×10^-6^ |
| DAF | CD55 | 0.29 | 4.98×10^-9^ | 3.87×10^-6^ |

## Table S3 Variable importance in projection (VIP) scores derived from OPLS-DA and differential analysis result in the positive ion mode.

| Metabolite Name | VIP | Fold change | p-value |
| --- | --- | --- | --- |
| Acetylcarnitine | 45.44 | 0.60 | 3.71×10^-5^ |
| L-carnitine | 23.76 | 0.75 | 2.44×10^-4^ |
| Niacinamide | 23.02 | 1.20 | 6.37×10^-3^ |
| Phosphocholine | 18.82 | 1.20 | 0.13 |
| 1-hexadecanoyl-2-octadecadienoyl-sn-glycero-3-phosphocholine | 17.41 | 0.72 | 5.16×10^-4^ |
| Hypoxanthine | 13.91 | 1.09 | 0.36 |
| 1-palmitoyl-sn-glycero-3-phosphocholine | 12.86 | 0.88 | 0.24 |
| Histamine | 12.39 | 0.48 | 9.75×10^-4^ |
| 3-aminopyridine | 11.53 | 0.43 | 6.17×10^-4^ |
| Oleamide | 11.34 | 1.02 | 0.68 |

## Table S4 Variable importance in projection (VIP) scores derived from OPLS-DA and differential analysis result in the negative ion mode.

| Metabolite Name | VIP | Fold change | p-value |
| --- | --- | --- | --- |
| Arachidonic acid (peroxide free) | 19.69 | 1.28 | 9.48×10^-3^ |
| Linoleic acid | 16.53 | 0.98 | 0.81 |
| Palmitic acid | 15.10 | 1.01 | 0.92 |
| Cholesteryl sulfate | 14.59 | 0.59 | 0.13 |
| Oleic acid | 11.83 | 1.28 | 0.04 |
| (2-aminoethoxy)[3-[hexadec-1-en-1-yloxy]-2-[icosa-5.8.11.14-tetraenoyloxy]propoxy]phosphinic acid | 11.55 | 0.58 | 0.09 |
| Cis-4,7,10,13,16,19-docosahexaenoic acid | 11.42 | 1.69 | 1.26×10^-2^ |
| 7Z, 10Z, 13Z, 16Z, 19Z-Docosapentaenoic acid | 10.91 | 2.67 | 2.03×10^-4^ |
| Adrenic Acid | 10.85 | 2.44 | 1.36×10^-4^ |
| Pg 44:12 | 9.73 | 1.93 | 0.02 |

## Table S5 Clinical follow-up information of our cohort.

| ID | Age | Gender | Pathological subtype | Smoking | Tumor size (cm) | Surgery time | Recurrence time | Death time | PFS | OS | Treatment | Solid component (50% stratification) |
| --- | --- | --- | --- | --- | --- | --- | --- | --- | --- | --- | --- | --- |
| S1 | 51 | F | APA | No | 1-2 | 2021.9 | No | No | 5 years | 5 years |  |  |
| S2 | 62 | F | SPA | No | 2-3 | 2021.9 | 2024.10 | No | 3 years | 5 years | Osimertinib |  |
| S4 | 58 | M | SPA | Yes | 2-3 | 2021.9 | No | No | 5 years | 5 years |  |  |
| S5 | 58 | M | APA | Yes | 1-2 | 2021.10 | No | No | 5 years | 5 years |  |  |
| S6 | 41 | M | SPA | Yes | 2-3 | 2021.10 | 2023.8 | No | 22 months | 5 years | Fumateinib |  |
| S7 | 68 | M | PPA | Yes | 1-2 | 2021.10 | No | No | 5 years | 5 years |  |  |
| S8 | 66 | M | SPA | Yes | 1-2 | 2021.10 | No | No | 5 years | 5 years |  |  |
| S9 | 63 | F | SPA | Yes | 2-3 | 2021.10 | 2023.2 | No | 16 months | 5 years | Lorlatinib combined with radiotherapy |  |
| S10 | 63 | F | PPA | No | 2-3 | 2021.10 | 2025.7 | No | 45 months | 5 years | Chemotherapy and radiotherapy + targeted therapy |  |
| S12 | 57 | M | PPA | Yes | 1-2 | 2021.10 | No | No | 5 years | 5 years |  |  |
| S13 | 71 | M | APA | Yes | 1-2 | 2021.10 | No | No | 5 years | 5 years |  |  |
| S14 | 71 | F | APA | No | 2-3 | 2021.10 | 2024.4 | No | 30 months | 5 years |  |  |
| S15 | 67 | M | APA | Yes | 2-3 | 2021.11 | No | No | 5 years | 5 years |  |  |
| S16 | 75 | F | PPA | No | 2-3 | 2021.11 | No | No | 5 years | 5 years |  |  |
| S17 | 51 | F | PPA | No | 2-3 | 2021.11 | 2024.8 | No | 33 months | 5 years | Osimertinib |  |
| S19 | 75 | M | SPA | Yes | 2-3 | 2021.11 | No | No | 5 years | 5 years |  |  |
| S20 | 63 | M | SPA | Yes | 2-3 | 2021.11 | 2023.3 | No | 14 months | 5 years | radiotherapy |  |
| S21 | 75 | M | SPA | Yes | 2-3 | 2021.11 | No | No | 5 years | 5 years |  |  |
| S22 | 45 | F | APA | No | 0-1 | 2021.11 | No | No | 5 years | 5 years |  |  |
| S23 | 51 | F | APA | No | 2-3 | 2021.11 | 2022.7 | 2023.10 | 8 months | 23 months |  |  |
| S25 | 72 | F | SPA | Yes | 2-3 | 2021.11 | No | No | 5 years | 5 years |  |  |
| S26 | 47 | M | PPA | No | 2-3 | 2021.11 | No | No | 5 years | 5 years |  |  |
| S28 | 75 | F | APA | No | 2-3 | 2021.11 | No | No | 5 years | 5 years |  |  |
| S30 | 49 | F | IMA | No | 1-2 | 2021.11 | No | No | 5 years | 5 years |  |  |
| G1 | 65 | M | APA | Yes | 1-2 | 2021.9 | No | No | 5 years | 5 years |  | + |
| G2 | 53 | M | APA | Yes | 2-3 | 2021.9 | No | No | 5 years | 5 years |  | - |
| G3 | 68 | F | LPA | No | 1-2 | 2021.10 | No | No | 5 years | 5 years |  | - |
| G4 | 41 | F | MIA | No | 0-1 | 2021.10 | No | No | 5 years | 5 years |  | - |
| G5 | 31 | M | APA+IMA | No | 1-2 | 2021.10 | No | No | 5 years | 5 years |  | + |
| G6 | 32 | F | PPA | No | 0-1 | 2021.10 | No | No | 5 years | 5 years |  | - |
| G7 | 57 | F | APA | No | 1-2 | 2021.10 | No | No | 5 years | 5 years |  | - |
| G9 | 59 | F | MIA | No | 0-1 | 2021.10 | No | No | 5 years | 5 years |  | - |
| G10 | 66 | F | LPA | No | 0-1 | 2021.10 | No | No | 5 years | 5 years |  | - |
| G11 | 69 | F | LPA | No | 1-2 | 2021.10 | No | No | 5 years | 5 years |  | - |
| G12 | 44 | M | MIA | No | 0-1 | 2021.10 | No | No | 5 years | 5 years |  | - |
| G13 | 30 | F | MIA | No | 1-2 | 2021.10 | No | No | 5 years | 5 years |  | - |
| G14 | 61 | F | MIA | No | 0-1 | 2021.10 | No | No | 5 years | 5 years |  | - |
| G15 | 65 | F | PPA | No | 1-2 | 2021.10 | No | No | 5 years | 5 years |  | + |
| G16 | 46 | F | APA | No | 2-3 | 2021.10 | No | No | 5 years | 5 years |  | + |
| G17 | 62 | F | LPA | No | 1-2 | 2021.10 | No | No | 5 years | 5 years |  | + |
| G18 | 48 | F | MIA | No | 1-2 | 2021.10 | No | No | 5 years | 5 years |  | - |
| G19 | 62 | F | APA | No | 2-3 | 2021.10 | No | No | 5 years | 5 years |  | + |
| G20 | 54 | M | APA | Yes | 1-2 | 2021.10 | No | No | 5 years | 5 years |  | + |
| G21 | 68 | F | MIA | No | 2-3 | 2021.10 | No | No | 5 years | 5 years |  | - |
| G22 | 49 | F | APA | No | 2-3 | 2021.10 | No | No | 5 years | 5 years |  | + |
| G23 | 65 | F | APA | Yes | 1-2 | 2021.10 | No | No | 5 years | 5 years |  | + |
| G24 | 73 | F | PPA | No | 2-3 | 2021.10 | No | No | 5 years | 5 years |  | + |
| G25 | 48 | F | APA | No | 1-2 | 2021.10 | No | No | 5 years | 5 years |  | + |
| G27 | 67 | M | APA | Yes | 1-2 | 2021.11 | No | No | 5 years | 5 years |  | - |
| G31 | 67 | M | APA | Yes | 2-3 | 2021.11 | No | No | 5 years | 5 years |  | + |

# References

1.	Zhu Y, Xu H, Chen H, et al. Proteomic analysis of solid pseudopapillary tumor of the pancreas reveals dysfunction of the endoplasmic reticulum protein processing pathway. *Mol Cell Proteomics*. Oct 2014;13(10):2593-603. doi:10.1074/mcp.M114.038786

2.	UniProt C. UniProt: a worldwide hub of protein knowledge. *Nucleic Acids Res*. Jan 8 2019;47(D1):D506-D515. doi:10.1093/nar/gky1049

3.	Gotz S, Garcia-Gomez JM, Terol J, et al. High-throughput functional annotation and data mining with the Blast2GO suite. *Nucleic Acids Res*. Jun 2008;36(10):3420-35. doi:10.1093/nar/gkn176

4.	Quevillon E, Silventoinen V, Pillai S, et al. InterProScan: protein domains identifier. *Nucleic Acids Res*. Jul 1 2005;33(Web Server issue):W116-20. doi:10.1093/nar/gki442

5.	Kanehisa M, Sato Y, Morishima K. BlastKOALA and GhostKOALA: KEGG Tools for Functional Characterization of Genome and Metagenome Sequences. *J Mol Biol*. Feb 22 2016;428(4):726-731. doi:10.1016/j.jmb.2015.11.006

6.	Szklarczyk D, Kirsch R, Koutrouli M, et al. The STRING database in 2023: protein-protein association networks and functional enrichment analyses for any sequenced genome of interest. *Nucleic Acids Res*. Jan 6 2023;51(D1):D638-D646. doi:10.1093/nar/gkac1000

7.	Shannon P, Markiel A, Ozier O, et al. Cytoscape: a software environment for integrated models of biomolecular interaction networks. *Genome Res*. Nov 2003;13(11):2498-504. doi:10.1101/gr.1239303

8.	Chin CH, Chen SH, Wu HH, Ho CW, Ko MT, Lin CY. cytoHubba: identifying hub objects and sub-networks from complex interactome. 10.1186/1752-0509-8-S4-S11. *BMC Syst Biol*. 2014;8 Suppl 4(Suppl 4):S11. doi:10.1186/1752-0509-8-S4-S11

9.	Bader GD, Hogue CW. An automated method for finding molecular complexes in large protein interaction networks. *BMC Bioinformatics*. Jan 13 2003;4:2. doi:10.1186/1471-2105-4-2

10.	Csárdi G, Nepusz T. The igraph software package for complex network research. 2006:

11.	Xie Z, Bailey A, Kuleshov MV, et al. Gene Set Knowledge Discovery with Enrichr. *Curr Protoc*. Mar 2021;1(3):e90. doi:10.1002/cpz1.90

12.	Langfelder P, Horvath S. WGCNA: an R package for weighted correlation network analysis. *BMC Bioinformatics*. Dec 29 2008;9:559. doi:10.1186/1471-2105-9-559

13.	Aviram R, Manella G, Kopelman N, et al. Lipidomics Analyses Reveal Temporal and Spatial Lipid Organization and Uncover Daily Oscillations in Intracellular Organelles. *Mol Cell*. May 19 2016;62(4):636-48. doi:10.1016/j.molcel.2016.04.002

14.	Blazenovic I, Kind T, Ji J, Fiehn O. Software Tools and Approaches for Compound Identification of LC-MS/MS Data in Metabolomics. *Metabolites*. May 10 2018;8(2)doi:10.3390/metabo8020031

15.	Thevenot EA, Roux A, Xu Y, Ezan E, Junot C. Analysis of the Human Adult Urinary Metabolome Variations with Age, Body Mass Index, and Gender by Implementing a Comprehensive Workflow for Univariate and OPLS Statistical Analyses. *J Proteome Res*. Aug 7 2015;14(8):3322-35. doi:10.1021/acs.jproteome.5b00354

16.	Parkhomchuk D, Borodina T, Amstislavskiy V, et al. Transcriptome analysis by strand-specific sequencing of complementary DNA. *Nucleic Acids Res*. Oct 2009;37(18):e123. doi:10.1093/nar/gkp596

17.	Mortazavi A, Williams BA, McCue K, Schaeffer L, Wold B. Mapping and quantifying mammalian transcriptomes by RNA-Seq. *Nat Methods*. Jul 2008;5(7):621-8. doi:10.1038/nmeth.1226

18.	Pertea M, Kim D, Pertea GM, Leek JT, Salzberg SL. Transcript-level expression analysis of RNA-seq experiments with HISAT, StringTie and Ballgown. *Nat Protoc*. Sep 2016;11(9):1650-67. doi:10.1038/nprot.2016.095

19.	Liao Y, Smyth GK, Shi W. featureCounts: an efficient general purpose program for assigning sequence reads to genomic features. 10.1093/bioinformatics/btt656. *Bioinformatics*. Apr 1 2014;30(7):923-30. doi:10.1093/bioinformatics/btt656

20.	Benjamini Y, Yekutieli D. The control of the false discovery rate in multiple testing under dependency. *The Annals of Statistics*. 2001;29(4):24. doi:10.1214/aos/1013699998

21.	Yu G, Wang LG, Han Y, He QY. clusterProfiler: an R package for comparing biological themes among gene clusters. *OMICS*. May 2012;16(5):284-7. doi:10.1089/omi.2011.0118

22.	Rohart F, Gautier B, Singh A, Le Cao KA. mixOmics: An R package for 'omics feature selection and multiple data integration. *PLoS Comput Biol*. Nov 2017;13(11):e1005752. doi:10.1371/journal.pcbi.1005752

23.	Karnovsky A, Weymouth T, Hull T, et al. Metscape 2 bioinformatics tool for the analysis and visualization of metabolomics and gene expression data. 10.1093/bioinformatics/btr661. *Bioinformatics*. Feb 1 2012;28(3):373-80. doi:10.1093/bioinformatics/btr661
